# Translational Genomics and Community‐Driven Research in Australia

**DOI:** 10.1002/eahr.60031

**Published:** 2025-09-27

**Authors:** Fiona Russo, Keri Finlay, Isabella Sherburn, Tiffany Boughtwood, Jane Tiller

**Affiliations:** ^1^ Senior fellow at the Centre for Health Research at the University of Southern Queensland; ^2^ Program coordinator at Australian Genomics at the Murdoch Children's Research Institute; ^3^ Project officer at Australian Genomics at the Murdoch Children's Research Institute; ^4^ Managing director of Australian Genomics at the Murdoch Children's Research Institute; ^5^ Ethical, legal, and social advisor in public health genomics at the School of Public Health and Preventive Medicine at Monash University

**Keywords:** community involvement, community engagement, genomics, genetics, translational research, Australia, consumer involvement

## Abstract

The need for community involvement in human translational research has gained international recognition, with a growing consensus on its critical role in ensuring ethical and impactful health outcomes. In Australia, several bodies are beginning to mandate community involvement in research, reflecting this global trend. However, there remains a notable gap in practical guidance on effectively engaging communities, particularly in the emerging field of genomic research. To address this, Australian Genomics, a national collaboration supporting the translation of genomic research into clinical practice, coordinated an initiative called Involve Australia (IA). IA has developed comprehensive guidelines, together with and informed by the needs and perspectives of those directly impacted by genomic research, through surveys, interviews, and public consultations. The IA Guidelines provide practical information for genomic researchers on involving community members effectively and meaningfully in projects to ensure effective research translation, emphasizing the importance of building relationships, setting clear expectations, valuing community contributions, evaluating and reporting on the community involvement process, and translation of research outcomes. The IA Guidelines have been endorsed by various research and patient organizations, demonstrating the growing support for resources in this area. The development and implementation of these guidelines represent a crucial step forward in promoting community involvement in genomic research, setting a precedent for other areas of health and medical research to follow.

Community involvement (also known as consumer, patient, and public involvement) in health‐related research enhances research quality and supports effective research translation.^1^ It promotes trust and transparency by aligning research with community needs and embedding in research studies principles of ethics, equity, and accessibility. Community involvement in research also holds researchers and institutions accountable to the public through shared design and decision‐making, which in turn improves participant recruitment and retention and quality of research. When community involvement is aimed toward practical application and meaningful improvement in health care delivery and policy‐making, it supports better health outcomes.^2^ Translational health research aims to implement research findings into real‐world clinical contexts for sustainable health improvements. The field of genomic medicine, still in the initial stages of integration into clinical practice, can be effectively translated into public health outcomes through meaningful inclusion of community voices.

## COMMUNITY INVOLVEMENT IN HEALTH RESEARCH: A DEVELOPING INTERNATIONAL PRIORITY

Despite compelling evidence of the value of community involvement in research, it is not universally mandated by national and international funding bodies. However, some health research organizations and funders are beginning to encourage or require aspects of community involvement. The World Health Organization (WHO) emphasizes the importance of community engagement in research and translation, particularly for global health initiatives, noting that it “enables changes in behavior, environments, policies, programs and practices within communities.”^3^

Horizon Europe, the European Union's key research funding program, promotes “citizen engagement” within its call for broader public participation in research, but stops short of imposing strict requirements.^4^ Some countries within the EU, like the Netherlands^5^ and Denmark,^6^ have strong national policies supporting public involvement in health research. In the United Kingdom, the National Institute for Health and Care Research requires patient and public involvement (PPI) in funded research.^7^ At the institutional level, human research ethical approvals in the U.K. often require a demonstration of meaningful community engagement.^8^ In the United States, the Patient‐Centered Outcomes Research Institute mandates patient and stakeholder involvement in funded projects.^9^ The Canadian Institutes of Health Research promotes integrated knowledge translation, which involves patients and communities throughout the research process.^10^ In Japan, the Agency for Medical Research and Development has been at the forefront of promoting PPI^11^ and has developed a PPI guidebook^12^ to facilitate effective collaboration between researchers and patients, aiming to bring medical research outcomes to practical use more quickly.

Consumer involvement in health research varies across African countries, with some nations developing specific guidelines to promote ethical and participatory practices. For example, South Africa has established comprehensive guidelines emphasizing consumer involvement in health research.^13^ These guidelines underscore the importance of community engagement, advocating for the inclusion of community representatives in research processes to ensure ethical standards and cultural sensitivity.^14^

## COMMUNITY INVOLVEMENT IN AUSTRALIAN RESEARCH

The National Health and Medical Research Council, Australia's leading public health research funding body, requires consumer and community involvement in some of the research it funds, and their *Statement on Consumer and Community Involvement in Health and Medical Research* outlines best practices for meaningful engagement.^15^ Projects funded through Australia's government‐funded Medical Research Future Fund are expected to demonstrate consumer and community involvement.^16^ The Australian Code for the Responsible Conduct of Research^17^ applies to all publicly funded research and requires transparency and engagement with those affected by the research. Some Australian jurisdictions have specific policies mandating consumer involvement in health research and service design, such as “WA Health and Medical Research Strategy 2023‐2033.”^18^ While not every Australian jurisdiction requires community involvement, many funding opportunities are contingent upon meaningful engagement with consumers.

## GENOMIC RESEARCH PRIORITIES

Genomic medicine is enabling innovation that raises ethically complex issues requiring community involvement. A 2019 report estimated that more than 60 million individuals internationally would have their DNA sequenced by 2025.^19^ Genomics has assisted the development of diagnostics, medicines, and therapies for a variety of health issues including cancer and rare disease.^20^ A 2019 review found more than 96 genomic initiatives had been reported internationally.^21^ In a rapidly evolving landscape, it is critical that community members are meaningfully and responsibly involved in research design and translation efforts, to ensure equity as genomic medicine continues to transform health care.^22^ However, Jack Nunn et al. conclude that only 33% of identified initiatives reported to the public on community involvement.^23^

## INVOLVE AUSTRALIA

Australian Genomics^24^ is a government‐funded, national research collaboration focused on integrating genomics into health care and aims to create a more inclusive and effective research environment. Involve Australia (IA) is a community‐led initiative of Australian Genomics that stemmed from the need for greater public engagement in genomic research,^25^ recognizing the importance of involving patients, families, advocacy groups, and other key stakeholders in shaping this growing area of research. IA was loosely modelled on INVOLVEUK, a government‐funded program that promoted public involvement in research and has since been subsumed into the National Institute for Health and Care Research.^26^ IA promotes the principles of involvement by collaborating with patient support and advocacy groups, patients, caregivers, researchers, and health care professionals to encourage and support effective community engagement and involvement in genomic research.

IA is supported by Australian Genomics but led by an expert working group comprising patient advocates, leaders of genetic patient support and advocacy groups, and researchers. In a community‐driven effort, the concept of IA was first raised with Australian Genomics by a patient support and advocacy group leader. The two groups worked together to enlist experienced patient advocates, community involvement experts, and researchers to form a working group. A snowball‐type approach was used where invitations to participate were distributed to people known to already recruited working group members. Some members of the working group held dual roles. The first IA meeting was held in July of 2021. In June of 2022, community members on the working group were offered an honorarium upon recommendation by a group member.

This collaborative framework ensured that the diverse perspectives of patients, advocates, clinicians, researchers, and institutions were integrated into the development of IA's first publication, *Guidelines for Community Involvement in Genomic Research* (IA Guidelines).^27^ The IA Guidelines provide practical information and guidance for researchers seeking to involve community members meaningfully in their projects, including ideation, design, delivery, and translation of health research, particularly in the field of genomics.

By bringing together stakeholders from various backgrounds in their development, the IA Guidelines foster and model a comprehensive and inclusive approach to research. This approach contributes to the development of projects that are not only scientifically robust, but also relevant and acceptable to communities and ethically sound. The collaborative framework promotes transparency, trust, and mutual respect between researchers and the community, ultimately enhancing the quality and impact of genomic research in Australia.

## DEVELOPING THE GUIDELINES

### Data collection

The first stage involved conducting a scoping review to identify and analyze the content covered in pre‐existing Australian community involvement guidelines (n = 21) and to identify involvement methodologies described in national and international genomic research publications (n = 22). Pre‐existing guidelines often suggested that researchers address different priority areas of community involvement (e.g., remuneration, evaluating impact of involvement) but did not always describe how to undertake this in practice. Moreover, pre‐existing guidelines frequently provided little detail when reporting community involvement processes during the guidelines’ development, though the peer‐reviewed genomics literature provided more detail on involvement processes than other types of literature reviewed.

**A global shift toward community involvement in translational research is emerging, assisted by the strong encouragement or mandates of funding bodies and the evolution of research culture to recognize the importance of prioritizing community voices.**

The second stage of data collection involved surveying the Australian public's perspectives on community involvement in health‐related research activities.^28^ The survey was designed by the working group and aimed to document previous participation and involvement in research, and barriers and enablers to community involvement in health research. A market research company recruited 1,156 participants to complete the survey. Key findings highlighted a low level of community confidence in contributing to research, a limited understanding of community involvement, concerns regarding trust and governance of data, and some mistrust of research funders. However, more than 60% of participants reported an interest in research, believed research should be informed by community views, and wanted to have their voices heard.

Semistructured interviews (n = 20) formed the third stage of data collection. These identified barriers and enablers to community involvement from the perspectives of community members involved in research (n = 5), community involvement coordinators (n = 6), researchers who have worked with community members (n = 7), and institutional leads where community involvement programs were underway (n = 2). These interviews enabled exploration of community involvement drivers, strategies currently being undertaken, and distillation of learnings from those with experience in this area. While identifying areas where practical guidance for researchers could be implemented, the interviews also highlighted many systemic barriers that make community involvement challenging. These barriers included lack of funding and institutional/organizational policies to support community involvement, the time‐limited nature of research, and the dearth of guidance for remuneration of community members.

We found that when organizational leaders championed and resourced community involvement, it became part of the organizational culture, driving an expectation that all researchers would follow. Long‐term relationships between community members and researchers, and funding bodies with community involvement mandates, were also highlighted as driving community involvement in research. The final IA Guidelines cover several key domains to ensure effective and meaningful community engagement (see box 1).

**Box 1. Domains of Community Involvement in IA Guidelines**

1. ***Building relationships:*** Emphasizes the importance of establishing trust and mutual respect between researchers and community members. It includes strategies for initiating and maintaining these relationships throughout the research process, such as holding one‐on‐one informal meetings with community members.
2. ***Setting expectations:*** Focuses on clearly defining the roles, responsibilities, and expectations of all stakeholders involved. This helps in aligning goals and ensuring transparency in the research process.
3. ***Valuing community contributions:*** Highlights the significance of recognizing and appreciating the input and efforts of community members. This domain provides guidance on acknowledging and rewarding contributions appropriately.
4. ***Evaluating and reporting:*** Provides methods for assessing the effectiveness of community involvement and reporting the outcomes. This ensures continuous improvement and accountability in the engagement process and promotes building an evidence base for the impact of community involvement.
5. ***Translating research outcomes:*** Offers strategies for ensuring that research findings are communicated back to communities and stakeholders in meaningful and accessible ways. This includes translating complex scientific results into accessible information that can be used to benefit the community, and partnering with communities in advocacy efforts.

### Consultation with stakeholders

Following the completion of the initial draft, a public consultation was undertaken to ensure community voices were adequately reflected within the IA Guidelines, and that they met researcher needs. The IA Guidelines were reviewed by 46 individuals, including genomic and nongenomic researchers and health professionals, patients, community members, and patient support and/or advocacy groups. Respondents were overwhelmingly supportive of the IA Guidelines, with all drafted recommendations receiving at least 80% positive ratings. Recommendations that required amendment included those regarding prioritizing diverse voices in involvement and using trauma‐informed approaches, which were revised in accordance with stakeholder feedback.

### Impact of the working group

Consistent with our own values with respect to inclusive community involvement, the expertise of the working group and regular meeting discussions underpinned and were essential to the study design, analysis of the collected data, review of the consultation feedback, and drafting of the IA Guidelines. There was genuine leadership from the working group to set the direction, refine methodologies, contextualize data analysis, and collaboratively author the IA Guidelines. This process ensured that the IA Guidelines incorporated multiple perspectives, especially from those impacted by genomic research and its outcomes. The process of involving the working group members and the impacts from that involvement were publicly reported using Standardized Data on Initiatives.^29^

Once consultation feedback was incorporated, an additional review by a small number of community members (n = 4) was undertaken to ensure community voices remained prominent in the final version of the IA Guidelines. These community members were external to the working group and were offered remuneration for their contributions. The final IA Guidelines were published and made freely available via the Australian Genomics website^30^ in December of 2023, where they have been downloaded 359 times at the time of this writing. Australian researchers and institutions were encouraged to apply the IA Guidelines to enhance patient and public involvement in genomic research, with 23 patient support and advocacy and research organizations formally endorsing the IA Guidelines. This endorsement list is recorded on the Australia Genomics website.

## RECOMMENDATIONS TO FUNDERS AND INSTITUTIONS

In addition to the IA Guidelines, in May of 2024, IA also released recommendations for health and medical research funders^31^ and institutions^32^ outlining how they can support community involvement in research. The aim of these recommendations was to provide health and medical research funders and institutions with strategies to improve their community involvement processes, and support researchers and community members to work collaboratively in more effective and inclusive ways. These recommendations were devised by the IA working group, using their collective knowledge, and incorporating the insights and learnings gleaned from the data described above.

## TRANSLATIONAL IMPACT

The IA Guidelines and accompanying documents were designed to primarily benefit two key user groups: communities sharing their research involvement preferences and expectations, and researchers and institutions seeking practical advice on how to meaningfully engage with communities in the conduct of research. The translation of research outcomes into good clinical practice is facilitated by a strong researcher‐community partnership, particularly in identifying the barriers and enablers of access to health services for the community. The IA Guidelines offer explicit and practical advice for knowledge dissemination and translation into the community, extending their value beyond the immediate context of individual research projects.

To further measure the translational impact of the IA Guidelines, a study investigating the integration of the IA Guidelines into research activity is currently underway. In one case study, the IA Guidelines are being implemented by a project focusing on increasing genetic diagnoses in undiagnosed cohorts through functional testing. Community members who have been involved in the project from the early stages, and who informed study design through to project implementation, have also participated in this investigative follow‐up. Early findings from this follow‐up study suggest that use of the IA Guidelines has influenced the relationship between researchers and community members and led to community members feeling valued and comfortable enough to provide constructive feedback to the research team. Researchers have also reported feeling more capable of considering where community input would be most valuable. This has enabled the application of a truly participant‐centered approach throughout the project. Further follow‐up case studies are ongoing.

## CONCLUSION

A global shift toward community involvement in translational research is emerging, assisted by the strong encouragement or mandates of funding bodies and the evolution of research culture to recognize the importance of prioritizing community voices. The evidence shows that community involvement leads to better translational research outcomes, but practical guidance about how to effectively engage communities has been lacking. The IA Guidelines have been developed to facilitate equitable and responsible implementation of genomic research through the integration of community involvement. The IA Guidelines, and this paper, have provided a model for the consideration and development of guidance by international bodies and those in other areas of health and medical research.

## ACKNOWLEDGMENTS

The authors acknowledge the funding of Australian Genomics by a National Health and Medical Research Council of Australia grant.

## INVOLVE AUSTRALIA WORKING GROUP

Tiffany Boughtwood, John Cannings, Monica Ferrie, Keri Finlay, Anne McKenzie, Sean Murray, Jack Nunn, Greg Pratt, Fiona Russo, and Isabella Sherburn.
